# A Practical Treatise on Artificial Crown and Bridge-Work

**Published:** 1897-05

**Authors:** 


"A Practical Treatise on Artificial Crown- and Bridge-work." By Dr. George Evans, Fifth Edition, with 625 Illustrations. Publishers: S. S. White Dental Mfg. Co., Philadelphia.

This new edition of what has become an authority on crown- and bridge-work, merits all and more that has been said in its favor by reviewers of the former editions. To prevent a bulky presentation, the author in this fifth edition has omitted obsolete matter, curtailed descriptions of unimportant variations, avoided, as far as possible, repetitions, and endeavored to treat his subjects concisely, without sacrificing clearness for brevity. There is no doubt but that this new edition will receive the same hearty approval from the dental profession as the preceding ones.

